# Supplementary material for: Ultralight, Ultraflexible, Anisotropic, Highly Thermally Conductive Graphene Aerogel Films
Source: Molecules. 2021 Nov 14;26(22):6867. doi: 10.3390/molecules26226867 (PMC8623857; doi:10.3390/molecules26226867)
Supplement: Supplementary file 1 [file molecules-26-06867-s001.zip › molecules-1417040-supplementary.pdf]

Supplementary Information

Ultralight, ultraflexible, anisotropic highly thermally conductive graphene aerogel films

Zheng Liu[1,], Qinsheng Wang[1], Linlin Hou[2], Yingjun Liu[2], & Zheng Li[2]*

1 Special Equipment Safety Supervision and Inspection Institute of Jiangsu Province, National Graphene Products Quality Inspection and Testing Center (Jiangsu), 330 Yanxin road, Huishan, Wuxi, P. R. China., Jiangsu Province 214174, P. R. China; Liuzheng1027@163.com (Z.L.); wqs@wxtjy.com (Q.W.)

2 MOE Key Laboratory of Macromolecular Synthesis and Functionalization, Department of Polymer Science and Engineering, Key Laboratory of Adsorption and Separation Materials & Technologies of Zhejiang Province, Zhejiang University, 38 Zheda Road, Hangzhou 310027, P. R. China; yilunliu@mail.xjtu.edu.cn (Y.L.), lizheng_zju@zju.edu.cn(Z.L.)

* Correspondence: Liuzheng1027@163.com; Tel.:+86-(0)51083256600 (Z.L.)

Supporting information file includes:

Figures S1 to S5.

Tables S1.

Supporting Information

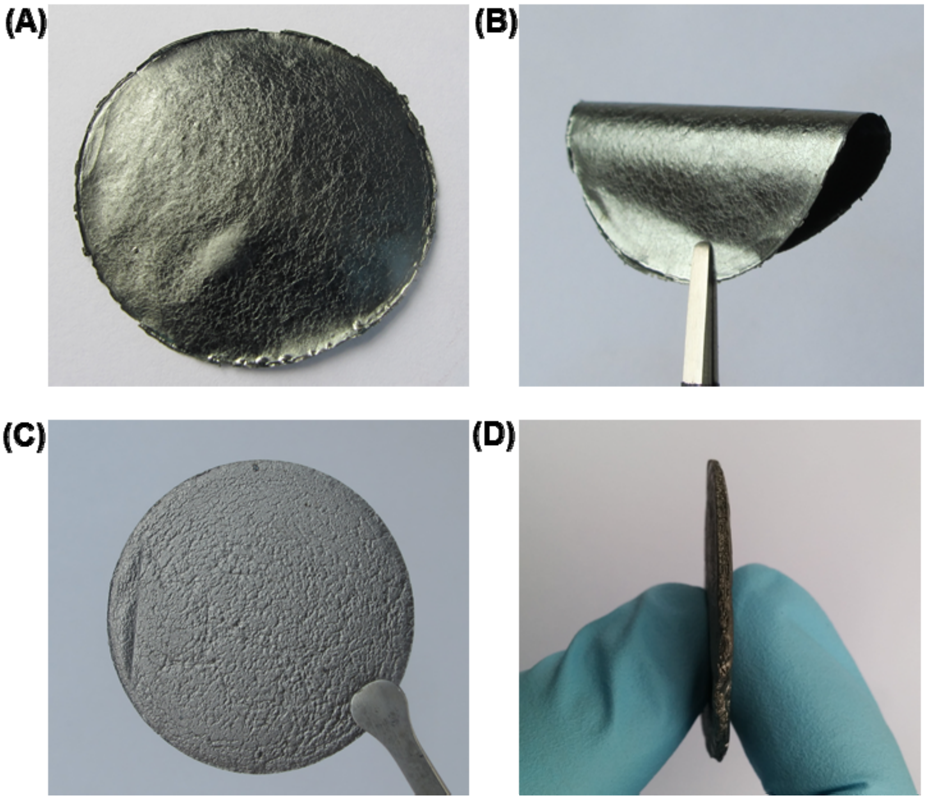

Figure S1. (**A**,**B**) Graphene film reduced by HI solution. (**C**,**D**) Graphene film after carbonization under 3000 ºC with loose and porous structure.

Table S1. Detailed data of Elemental analysis, XRD and Raman for GO and GAFs at different annealing temperature.

Sample	C(%)	O(%)	C+O	Peak 2θ	d-spacing	Crystal sizes	ID/IG	I2D/IG	D	G	2D
GO	46.86	44.249	91.109	/	/	/	0.79	0.065457	1362.32	1587.66	2693.7
GAF-HI	53.96	36.28	90.24	24.54	3.623	8.742	1.05	0.066844	1352.17	1576.97	2691.9
GAF500	62.76	31.182	93.942	25.35	3.509	7.441	0.78	0.024827	1354.25	1575.34	2698.42
GAF1000	90.08	3.63	93.71	26.16	3.402	11.108	1.02	0.079894	1351.41	1575	2701.7
GAF1500	92.55	0	92.55	26.32	3.382	12.875	0.16	0.258177	1360.27	1579.37	2717.3
GAF2000	95.12	0	95.12	26.37	3.376	13.597	0.03	0.40775	1360	1578.2	2723.18
GAF2500	99.69	0	99.69	26.48	3.362	26.985	0.004	0.339415	1375.91	1580.43	2740.2
GAF3000	100.37	0	100.37	26.52	3.357	27.984	0.004	0.321718	/	1578.29	2740.68

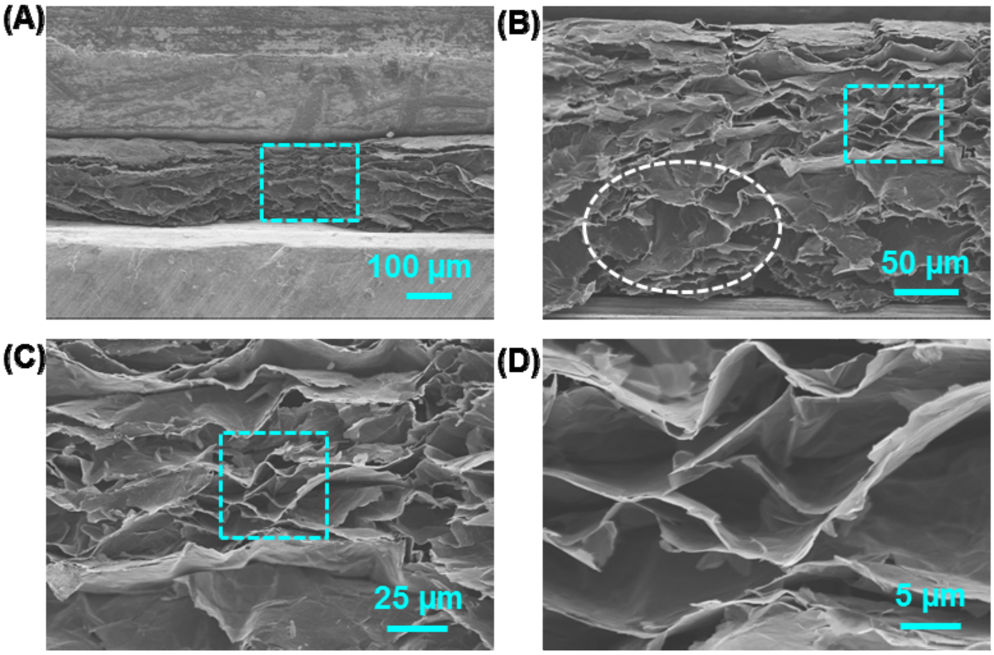

Figure S2. Cross-section of a GAF annealed at 3000 ºC with a loose and porous structure that made by tear opening.

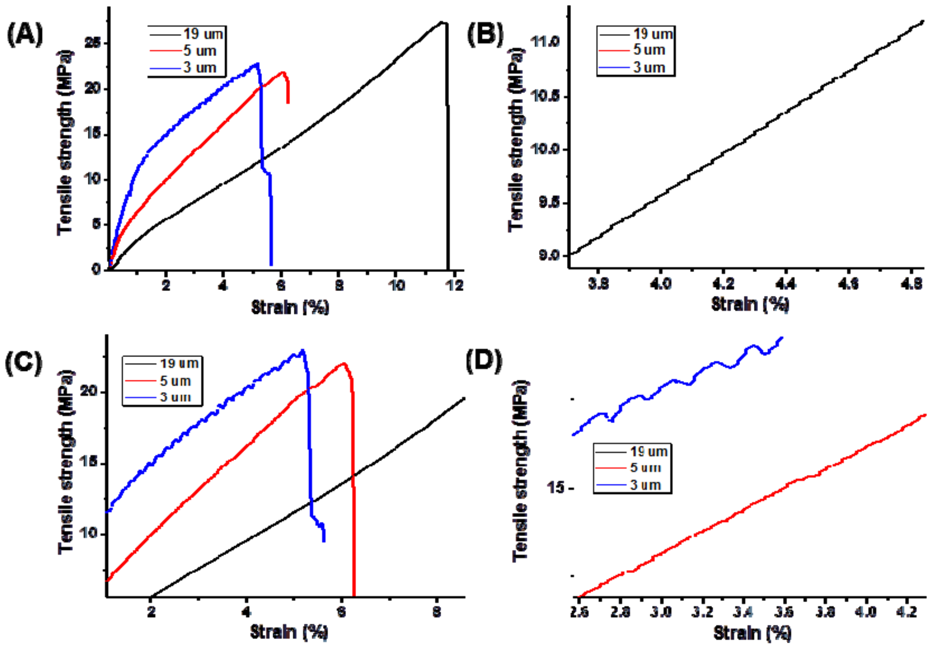

Figure S3. Stress-strain curves of compressed GAFs with thicknesses of 3 µm, 5 µm and 19 µm. All three curves performed obvious step-like periodic features, while the 19 µm film in a much smaller scale.

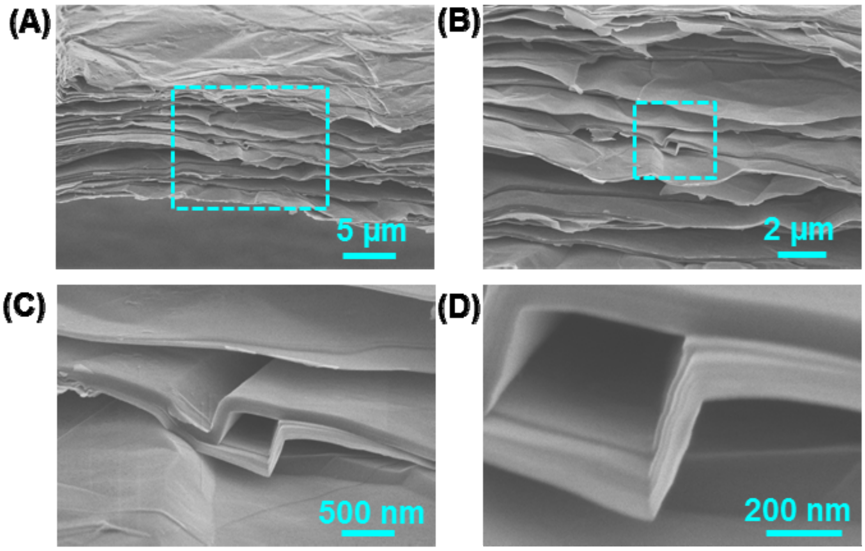

Figure S4. Cross-section of compressed GAFs that illustrates Z-shape folding formed by compression of multilayer graphene of gas chambers.

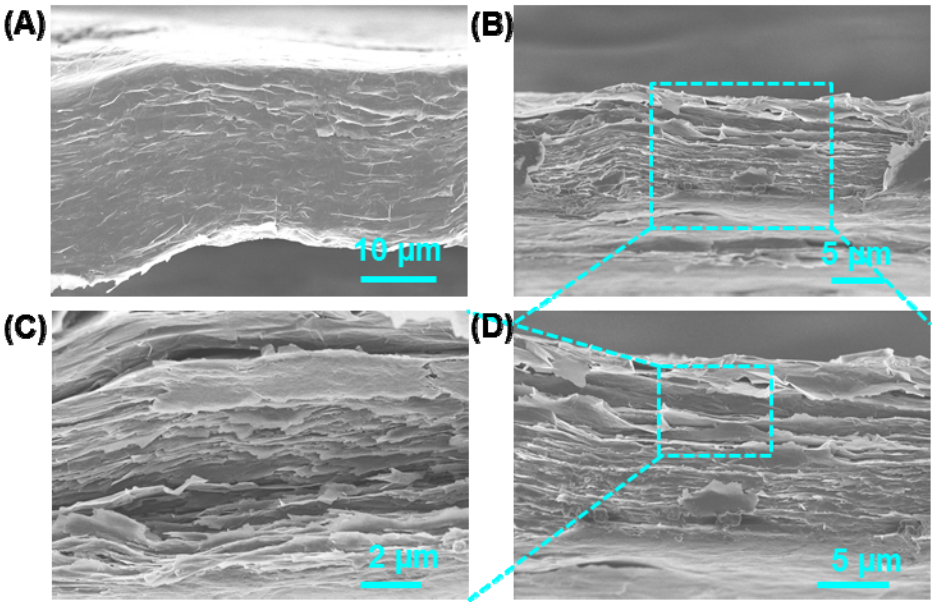

Figure S5. Cross-section of a pressed graphene film with highly compacted and oriented structure.
